# The correct typification of *Tradescantia crassula* (Commelinaceae)

**DOI:** 10.3897/phytokeys.80.13448

**Published:** 2017-06-05

**Authors:** Gustavo Hassemer, Luís A. Funez, João Paulo R. Ferreira, Lidyanne Y. S. Aona

**Affiliations:** 1 Statens Naturhistoriske Museum, Københavns Universitet, Sølvgade 83 S, 1307 Copenhagen, Denmark; 2 Herbário Dr. Roberto Miguel Klein (FURB), Universidade Regional de Blumenau, Campus I, 89012-900, Blumenau, SC, Brazil; 3 Laboratório de Moluscos Marinhos, Universidade Federal de Santa Catarina, Servidão Beco dos Coroas, 503, 88061-600, Florianópolis, SC, Brazil; 4 Centro de Ciências Agrárias, Ambientais e Biológicas, Universidade Federal do Recôncavo da Bahia, Rua Rui Barbosa, 710, 44380-000, Cruz das Almas, BA, Brazil

**Keywords:** Humboldt, Kunth, Link & Otto, Sello

## Abstract

Here, we present evidence that the alleged correction of the typification of *Tradescantia crassula* recently proposed by Pellegrini, Forzza and Sakuragui is erroneous. Furthermore, we clarify misconceptions concerning the epitype of *T. crassula*, the specimen B-100521014, which was collected by Friedrich Sello in southern Brazil, and is not original material for *T. crassula*.

## Introduction

In their recently published article, Pellegrini et al. (2017) (hereafter: PFS) claim to make corrections to the typification of *Tradescantia crassula* Link & Otto (Commelinaceae) done by Funez et al. (2016) (hereafter: FHF). The objectives of this note is to present evidence that PFS’s alleged “correction” is erroneous, and to clarify misconceptions concerning the epitype of *T. crassula*, the specimen B-100521014, which was collected by Friedrich Sello in southern Brazil, and is not original material for *T. crassula*.

In their nomenclatural notes on this species, PFS (pp. 7–8) start affirming that FHF “indicate that Pellegrini (2015) erroneously designated the specimen *Sellow 3033* (B100521014) as the lectotype for *T. crassula*”. This is not true, as FHF (p. 70) stated that “Pellegrini (2015) (a M.Sc. dissertation) incorrectly regarded the sheet kept in B (barcode B100521014) as the lectotype for the name *T. crassula*”. No mention of “correcting” a typification was made by FHF, and the use of the word “regarded”, instead of the more usual “designated”, had the purpose of leaving clear that FHF were aware that the M.Sc. dissertation in question (Pellegrini 2015) was not a validly published document.

**Figure 1.**
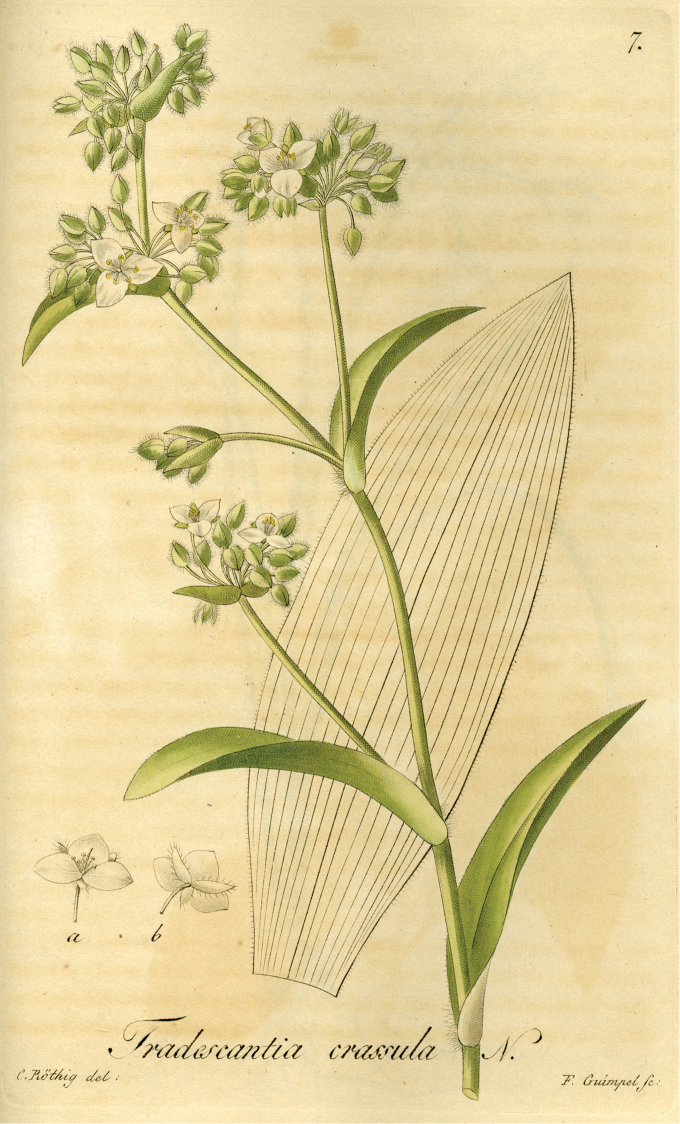
Lectotype of *Tradescantia crassula* (tab. 7 in Link and Otto 1828a), designated in Funez et al. (2016: 71).

Next, PFS argue that the collector of the specimen B-100521014 (see fig. 5 in Funez et al. 2016) should be considered to be Friedrich Sello (also known as Sellow, which is not his family name but is how he signed his name after 1814), and that the year written on the label, 1836, does not refer to the collection date of the specimen, but rather to the date when Karl Sigismund Kunth would have received it in Berlin (“We believe this date might correspond to the date when this specimen was acquired by Kunth, and placed into his personal herbarium”). On this basis, PFS proposed the designation of the specimen B-100521014 as the lectotype for the name *T. crassula* and, according to them, this newly designated lectotype would supersede the lectotype designated for this name in FHF (p. 71), i.e., tab. 7 in Link and Otto (1828a) (see Fig. 1)—“According to the *Code* (McNeill et al. 2012, Art. 9.2), the *Sellow 3033* (B100521014) specimen is a suitable choice for a lectotype, superseding the lectotypification of the original illustration, done by Funez et al. (2016)”.

Nevertheless, the interpretation of PFS of the type of *T. crassula* is erroneous. First of all, the authors cite the work that includes the protologue of *T. crassula* erroneously. The work cited, *Icones plantarum selectarum Horti Regii Botanici Berolinensis cum descriptionibus et colendi ratione* (Link and Otto 1828b), does not contain the description of *T. crassula*, which was in fact published in *Icones plantarum rariorum Horti Regii Botanici Berolinensis cum descriptionibus et colendi ratione*, vol. 1 (Link and Otto 1828a). The possibility that PFS have not read the protologue of *T. crassula* could perhaps explain the misunderstandings that these authors cause regarding the typification of this name.

**Figure 2.**
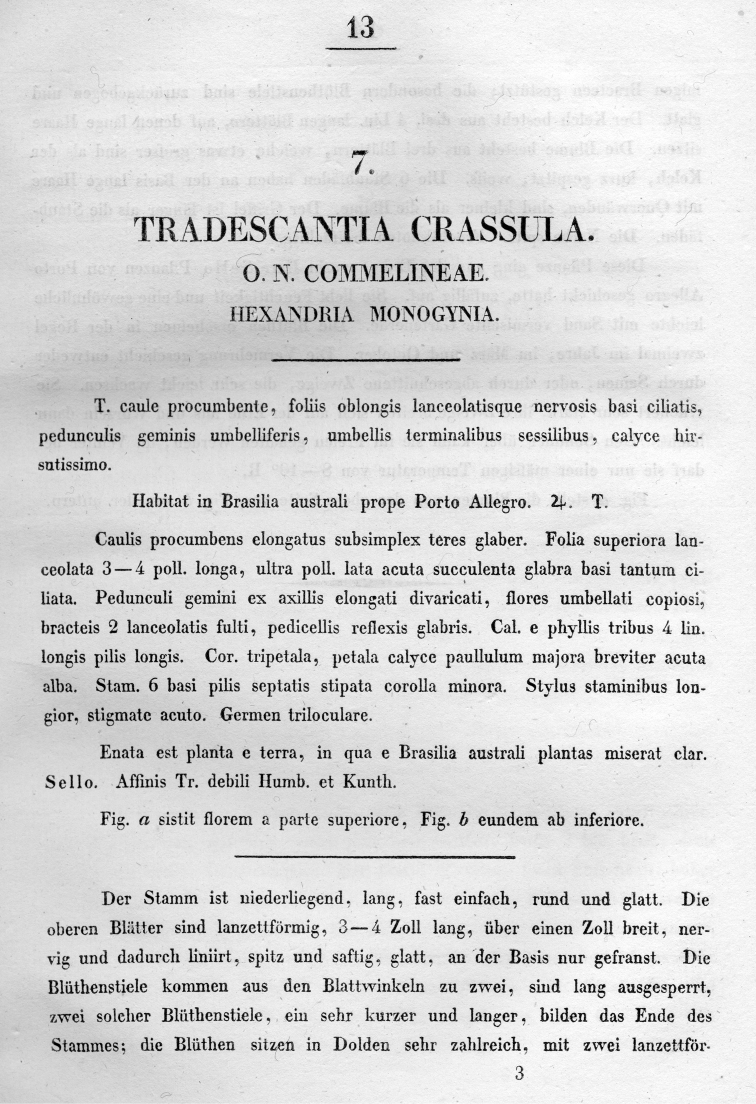
Protologue of *Tradescantia crassula*, page 1 (Link and Otto 1828a: 13).

**Figure 3.**
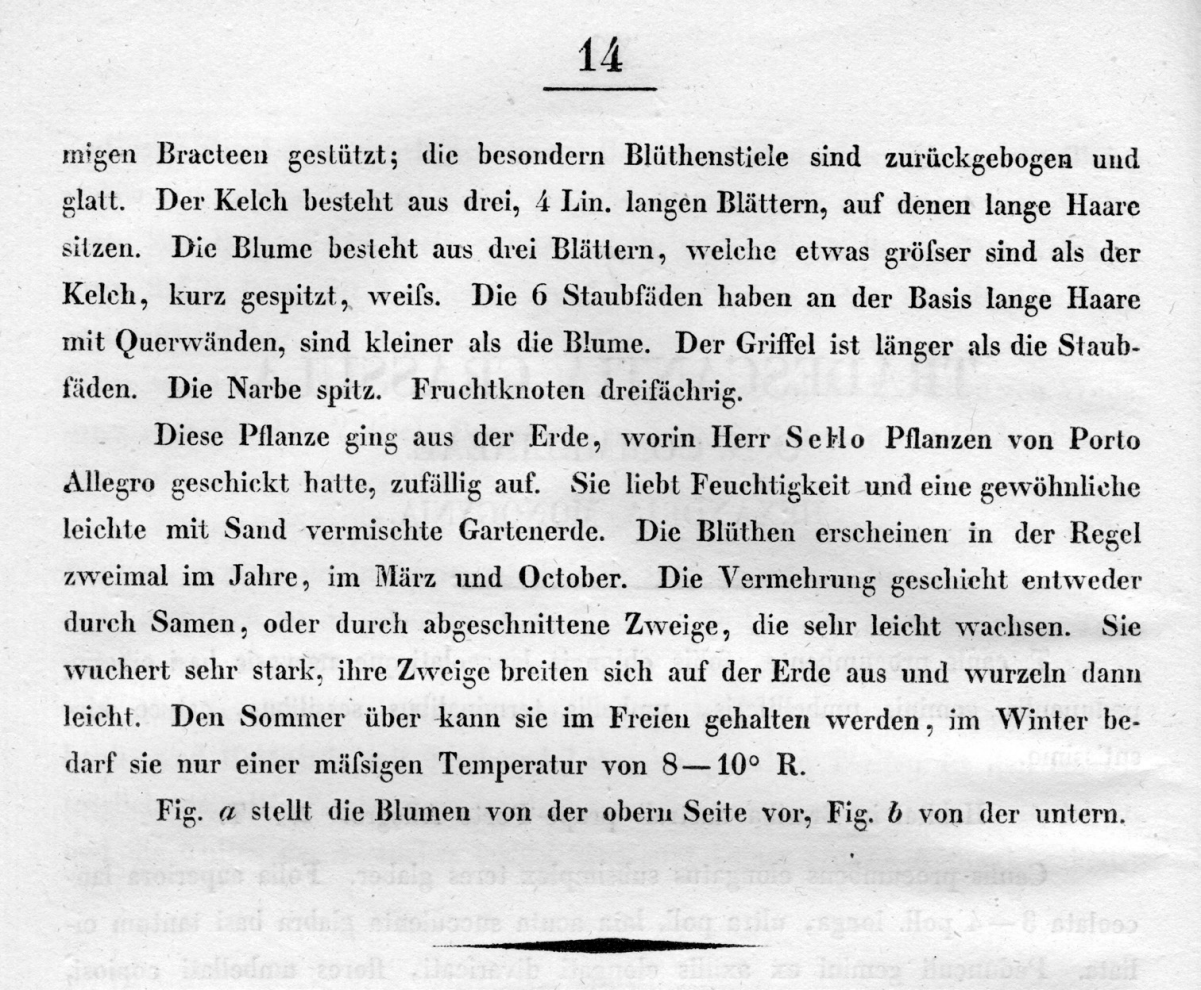
Protologue of *Tradescantia crassula*, page 2 (Link and Otto 1828a: 14).

A careful reading of the protologue of *T. crassula* (see Figs 2 and 3) leaves clear that this species was described based on plants which grew from seeds included by chance in the soil transported along with living plants sent by Sello from Porto Alegre (southern Brazil) to Berlin (“Diese Pflanze ging aus der Erde, worin Herr Sello Pflanzen von Porto Allegro geschickt hatte, zufällig auf” [This plant grew by chance from the earth with which Mr. Sello sent plants from Porto Alegre]). Further evidence for this is that the illustration (tab. 7) provided by Link and Otto (1828a) (see Fig. 1) is in colour, and clearly shows a living specimen, and not a dried herbarium specimen. Also, there is absolutely no evidence that either Heinrich Friedrich Link or Christoph Friedrich Otto have studied any herbarium specimens to describe *T. crassula* (see more details below). Therefore, since the type of *T. crassula* was cultivated in Germany, and not collected in Brazil, it is evident that it was not collected by Sello, a fact that invalidates the entire line of discussion conducted by PFS to try to invalidate the correct lectotypification done by FHF.

The specimen B-100521014 (*F. Sello 3033*), which was designated epitype of *T. crassula* by FHF (p. 71), has a complex history and has been the source of much misunderstanding. This specimen was collected by Sello in the then province of São Pedro do Rio Grande do Sul (today state of Rio Grande do Sul, southern Brazil) in November–December 1825 (Urban 1893; Marchiori et al. 2016), and originates from the set of duplicates which Alexander von Humboldt received in 1836 for his herbarium from Ignaz von Olfers and which he immediately passed on to Kunth, who kept the specimen in his personal herbarium until his death in 1850 (Urban 1893; Robert Vogt, pers. comm.). The label was most probably written by Kunth (Robert Vogt, pers. comm.); the text in the lower right part of the label reads “*Humboldt ded.* [dedit] *1836*” [donated by Humboldt in 1836], and not “Dec. 1836” as wrongly reported by both FHF and PFS (Robert Vogt, pers. comm.). This date, which is eight years after the description of *T. crassula*, refers to when Humboldt received this specimen, and not when it was collected. All things considered, the specimen B-100521014 clearly is not original material for *T. crassula*, and as a result the lectotypification and epitypification of this name proposed by FHF are correct and effective, in accordance to Arts. 9.2, 9.3, 9.8, 9.11, 9.12, 9.19, 9.20, 9.21, 9.22 and 9.23 of the Melbourne Code (McNeill et al. 2012).

On a final remark, PFS (p. 28) affirm that “many recent studies of Brazilian Commelinaceae have been narrowly focused, and proposed new species and several typifications (Funez et al. 2016; Hassemer et al. 2016a, 2016b; Hassemer 2017)” and that “perhaps the most unfortunate result of such studies is the potential for incorrect typification and application of names (e.g. Funez et al. 2016; Hassemer et al. 2016b; Hassemer 2017)”. Here, we must highlight that their critiques are completely unrelated to the content of their own article, since, with the exception of Funez et al. (2016), all works criticised (Hassemer et al. 2016a, 2016b; Hassemer 2017) dealt with the genus *Commelina* L., and not *Tradescantia* L. Therefore, these critiques can be regarded as gratuitous attacks, which are certainly not an example to be followed. Taxonomy advances best and fastest when taxonomists behave ethically, and collaborate, among themselves and with scientists of other fields.
